# Comparison of two active warming techniques on body temperature in healthy, anesthetized dogs premedicated with acepromazine or dexmedetomidine: A pilot study

**DOI:** 10.1371/journal.pone.0317997

**Published:** 2025-01-30

**Authors:** Jacob P. Rastas, Qianqian Zhao, Rebecca A. Johnson

**Affiliations:** 1 Department of Surgical Sciences, University of Wisconsin, Madison, Wisconsin, United States of America; 2 Department of Biostatistics and Medical Informatics, University of Wisconsin, Madison, Wisconsin, United States of America; Ross University School of Veterinary Medicine, SAINT KITTS AND NEVIS

## Abstract

Temperature regulation in dogs is significantly impaired during general anesthesia. Glabrous skin on paws may facilitate thermoregulation from this area and is a potential target for interventions attenuating hypothermia. This pilot study aimed to compare efficacy of an innovative warming device placed on the front paws (AVAcore; AVA), with no warming methods (NONE) and conventional truncal warming methods (CONV; circulating water blanket/forced air warmer) on rectal temperature and anesthetic recovery times. Dogs were premedicated with acepromazine (ACE) or dexmedetomidine (DEX), induced with intravenous propofol and maintained on isoflurane. The change in rectal temperature was statistically separated into three segments: 15 minutes following premedication, prior to induction (T0-T15), 15 minutes following anesthetic induction into isoflurane maintenance (T15-T30), and >30 minutes of isoflurane maintenance (>T30). Overall, when warming treatments and time points were combined, the decrease in rectal temperature from baseline was significantly greater with ACE than DEX (P < 0.05). When ACE and DEX were analyzed separately, changes in rectal temperatures did not differ between warming techniques at T0-T15 and T15-T30 (P > 0.05). However, at >T30 minutes, slopes of the temperature change differed between all three warming device groups, despite whether ACE or DEX was administered; temperature decreased least in CONV whereas the NONE had the largest decreases (P < 0.05). At >T30, when warming devices were considered separately, slopes of the temperature change in AVA and NONE did not differ between ACE and DEX (P > 0.050). However, in CONV, DEX had a significantly faster increase in slope than did ACE (P < 0.05). No differences in recovery times were observed between techniques or premedications (P > 0.05). Although CONV provided the most stable thermoregulation in anesthetized dogs, the AVAcore also moderated decreases in body temperature associated with general anesthesia despite premedication, providing an additional warming technique in dogs.

## Introduction

Mammalian thermal homeostasis is complex but is mainly regulated by hypothalamic inputs originating from the brainstem temperature, followed by core organ (brain, heart, lung, kidney, spleen) temperatures, and lastly skin temperature [1, 2]. However, thermoregulatory mechanisms are significantly impaired during inhalant anesthesia and inadvertent perianesthetic hypothermia (IPH) occurs in up to 85–89% of dogs. This is mainly due to loss of conscious thermoregulatory behaviors such as shivering, and through reduced autonomic responses in vasomotor tone and internal thermogenesis [3, 4]. In addition, select sedatives and anesthetics may cause vasodilation or inhibit vasoconstriction which contribute to heat loss. However, these play a small role in IPH compared with heat loss associated with autonomic blood flow tissue redistribution [5, 6].

Anesthetized patients lose heat via conduction to contacted objects, environmental radiation, evaporation, and convection via air or fluid movement between the body and environment or internally between tissues. During anesthesia, use of room temperature intravenous (IV) fluids, inhaled cold and dry gases, and cold preparatory solutions are associated with increased IPH through these mechanisms [6]. Although both passive (towels, blankets) and active (forced air warmers, circulating water blankets, etc.) heat conservation methods are frequently used, they may not be completely effective at maintaining thermal homeostasis [7–12]; some may even produce significant patient burns [13].

Rapid IPH develops during the initial stages of anesthesia, partially due to core heat redistributing to body surfaces due to anesthetic-induced inhibition of tonic thermoregulatory responses [7, 14, 15]. Thus, effective interventions to avoid or reduce IPH should likely be applied immediately following anesthetic induction. One common warming technique uses forced warm air applied to the trunk. However, although increased infection rates associated with this technique have not been shown in dogs [16], forced air warming is frequently not begun until surgical drapes are closely secured to the patient to reduce non-sterile particulates from blowing into the incision. This can delay therapeutic interventions until well into the hypothermic period. While external warming devices deliver heat to the truncal skin surface, vasoconstriction and high-resistance capillaries prevent heat from efficiently reaching the body core. Although active core warming techniques may be more efficacious in reducing IPH, they can be invasive and not clinically useful in most situations. Therefore, an innovative device that uses circulating warm water, the AVAcore, has been produced to target peripheral heat exchange arteriovenous anastomoses that bypass high-resistance capillaries (as defined by Pousielle’s Law) to quickly deliver a thermal load to the body core using circulating warm water. In humans, AVAcore placement on the glabrous (non-haired) skin of palms and feet containing arteriovenous anastomoses effectively restores normothermia in hypothermic post-surgical and cold-stressed healthy patients at a rate significantly faster than of conventional non-invasive methods [1, 17–19]. Canine paws, with their specialized glabrous (non-furred) skin, contain anastomoses like humans [20, 21]. However, vascular tone may affect heat regulation through these anastomoses and potentially AVAcore’s effectiveness when placed in these areas [13, 22]. Thus, the objective of these pilot studies was to assess the efficacy of the AVAcore in maintaining thermoregulation of anesthetized dogs sedated with premedications with different vasoactivity. We hypothesized that: 1) compared with no warming interventions and conventional warming methods (circulating water blanket and forced air warmer placed on the trunk), the AVAcore placed on both front paws at anesthetic induction will: 1) reduce hypothermia associated with general anesthesia similar to conventional warming methods, even when agents that differentially affect vascular tone are used (i.e. acepromazine, dexmedetomidine), and 2) expedite anesthetic recovery times.

## Materials and methods

### Animals

In consultation with a statistician, three healthy male beagles aged ~8 months old were used with mean body weight of 8.5±0.6 kg (mean±SD) in this crossover pilot study. They were deemed healthy by physical examination and normal packed cell volume, total protein, and blood urea nitrogen (Azostix; Siemens Healthcare Diagnostics Inc., Tarrytown, NY) values. The study was carried out with the recommendations in the Guide for the Care and Use of Laboratory Animals of the National Institutes of Health. The Institutional Animal Care and Use Committee in the School of Veterinary Medicine at the University of Wisconsin approved the protocol (Protocol Number: V006724). All efforts were made to ameliorate animal suffering.

Dogs were allowed to acclimate to their housing environment for 7 days prior to study start. Dogs were randomized using SAS Software (Cary NC) to receive three warming techniques in a crossover design following two anesthetic protocols.

### Warming techniques

**Fig 1:**
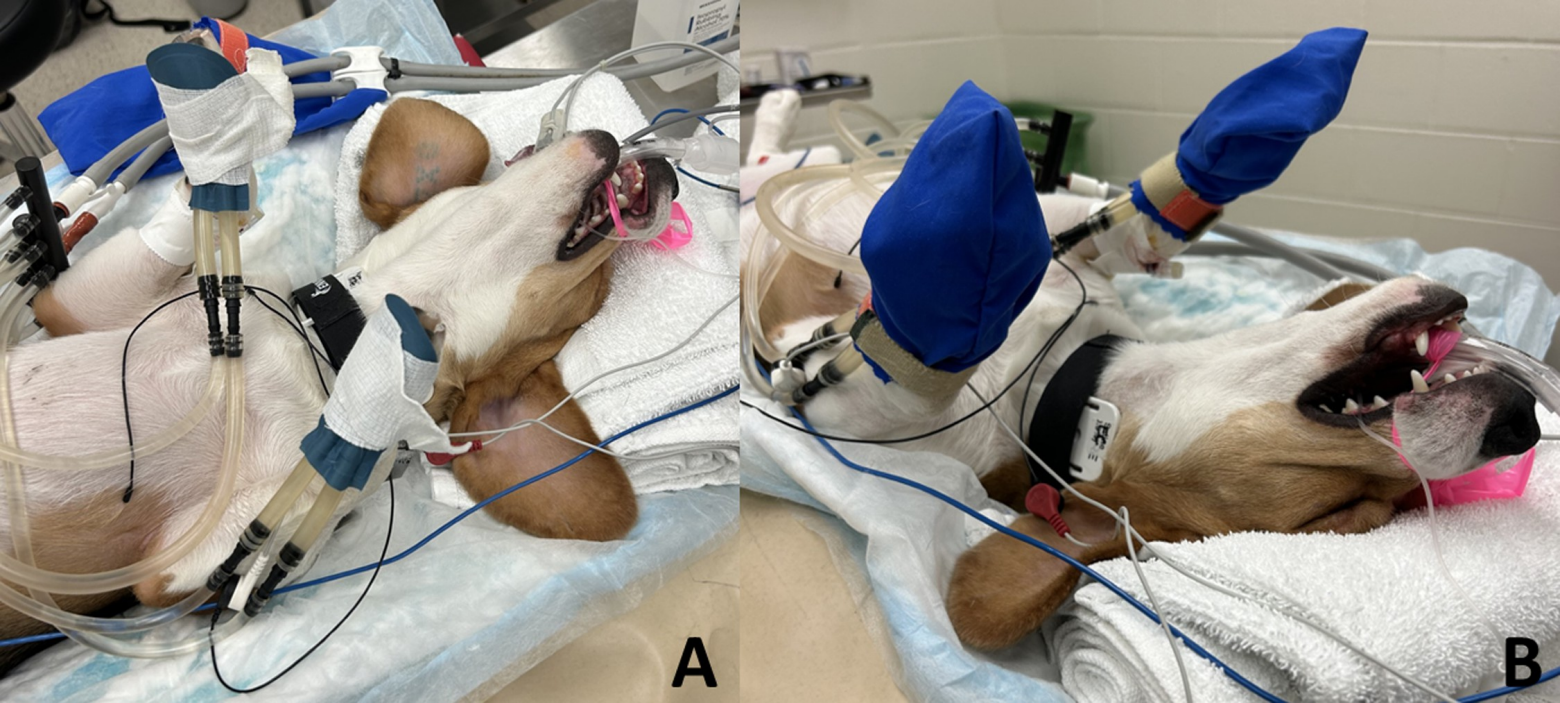
AVAcore placement. (A) Example of the AVAcore pads placed on both front paws of an anesthetized dog in dorsal recumbency, attached with an expansile wrap, and connected to the water pump. (B) AVAcore pads are then covered with a light, washable cotton pouch.

Warming devices used during experimentation included the AVAcore warming pads (AVAcore Technologies, Ann Arbor, MI) connected to a commercially available circulating water heating system (Gaymar T/Pump Classic, Gaymar Industries, Orchard Park, NY; 42°C) and placed on each front paw, attached with expansile wrap (mVet, Midwest Veterinary Supply, Lakeville, MN) and covered with a light cotton pouch (Fig 1), a large (75 X 56 cm) commercial circulating water blanket placed underneath each dog (Gaymar T/Pump Classic, Gaymar Industries, Orchard Park, NY; 42°C) and a forced air warmer blanket placed above each dog with the four corners secured with tape (Bair Hugger, Avante Animal Health, Louisville, KY; 40°C). In a randomized, cross-over design, following premedication with either acepromazine (ACE) or dexmedetomidine (DEX) (see Anesthetic protocol below), each dog had: 1) all warming devices placed but not turned on (NONE), 2) all warming devices placed but only conventional warming devices including the circulating warm water blanket and forced air warmer turned on (CONV), and 3) all warming devices placed but only the AVAcore turned on (AVA). Thus, each dog underwent 6 experiments (ACE-NONE, ACE-CONV, ACE-AVA, DEX-NONE, DEX-CONV, DEX-AVA) with at least 3 days between studies.

#### Anesthetic protocol

Clinically used premedications were chosen due to their potential effects on vascular tone. Each dog was sedated with either 1) acepromazine (0.05 mg/kg; ACE) intramuscularly to produce vasodilation via alpha-1 adrenergic receptor blockade [23] or 2) dexmedetomidine (5 mcg/kg; DEX) intramuscularly to produce vasoconstriction via alpha-2 adrenergic receptor activation [24], followed in 15 minutes by placement of a 22-gauge IV catheter in the right cephalic vein (Zoetis, Inc., Kalamazoo, MI). Doses were chosen based on clinical recommendations used at the primary institution. Propofol was administered IV at a rate of ~1 mL/20 seconds until loss of the palpebral reflex and sufficient jaw relaxation was noted to facilitate orotracheal intubation; amounts were recorded. Similar to previously published protocols investigating therapies to alleviate hypothermia [7, 11, 15, 25] general anesthesia was maintained using isoflurane delivered in 100% oxygen (~1 L/min) targeting an end tidal isoflurane (ETiso) of 1.3%. Mechanical ventilation was performed with a tidal volume of ~10 mL/kg, respiratory rate of 8–12 bpm, and peak inspiratory pressure of 8–10 cm H_2_O to maintain end tidal carbon dioxide (ETCO_2_) between 35–45 mmHg. Dogs were positioned in dorsal recumbency on a thin cotton pad covering a circulating water blanket and a #3 blood pressure cuff measuring ~40% of the limb circumference was placed just above the tarsus for systolic (SAP), diastolic (DAP) and mean blood pressure (MAP) measurement to indirectly estimate the degree of vasodilation or vasoconstriction. Animals were monitored with a pulse oximeter (SpO_2_), electrocardiogram (ECG), capnometer (ETCO_2_), and rectal thermometer (see specifics below) connected to a multiparameter monitor (Datascope, Passport 2, Mindray North America, Mahwah, NJ). Pulse rate, respiratory rate, SpO_2_, SAP, MAP and DAP, ETiso, ETCO_2_, and oxygen flow rate were recorded every 5 minutes, while rectal temperatures were recorded every 3 minutes. Room temperature was kept at 21.1–21.7°C (70–71°F) and unwarmed IV balanced crystalloids (Plasma-Lyte A, Baxter, Round Lake, IL) administered at 5 mL/kg/hr.

Immediately prior to premedication, a rectal thermometer (YSI 4600, YSI Incorporated, Yellow Springs, OH; accuracy = 0.115°C from 0–50°C) was inserted ~10 cm (4.5 inches) into the rectum, taped to the tail and temperature readings taken at baseline and throughout the experimental protocols including recovery. As previously detailed, all dogs had a circulating warm water blanket placed underneath, a forced-air warming blanket placed on top, and the AVAcore sleeves placed on the limbs immediately after anesthetic induction, with the appropriate devices turned on according to the treatment group assigned. Based on previously published reports [4, 19] dogs were maintained under anesthesia for ~2 hours, sufficient time to develop mild hypothermia, defined as a body temperature of <36.0°C. When a dog became moderately hypothermic (<35.0°C) or hyperthermic (>39.5°C), they were maintained for 10 additional minutes, then anesthesia discontinued, devices removed, and the recovery period began.

During recovery, dogs were placed undisturbed on an unheated floor pad until fully recovered and standing. Times to extubation, sternal recumbency and standing were recorded. Paws were inspected immediately after recovery and every day for 7 days for signs of irritation or injury (discoloration, swelling, licking, etc.).

### Statistical analyses

Statistical analyses were performed using SAS software version 9.4 (SAS Institute, Cary, NC). Since 3 dogs were repeatedly used in six treatment groups (ACE-NONE, ACE-AVA, ACE-CONV, DEX-NONE, DEX-AVA, DEX-CONV), the change in rectal temperature, heart rate, SAP, DAP, MAP, and recovery times (time to extubation, sternal recumbency and standing) were analyzed with a linear mixed-effect model using SAS PROC GLIMMIX. The linear mixed-effect model tested differences between treatments at each time point and potential interactions between time and treatment; normality was confirmed. Treatment groups were compared between ACE and DEX for similar warming device groups and between warming devices within the ACE and DEX groups. Temperature data (absolute change from baseline in rectal temperature) were analyzed using piecewise regression to account for the temperature following different linear time trends over three different time periods (T0-T15, T15-T30, >T30 minutes). For recovery times, the linear mixed-effect model was again used with only ACE/DEX groups and the final body temperature in the model. The Tukey-Kramer method was used for post-hoc comparisons. The amount of propofol required for adequate orotracheal intubation between ACE and DEX groups was compared with a one-way ANOVA followed by the Tukey post-hoc test. All P values were 2-sided, and P < 0.050 was used to indicate statistical significance. If the overall P value was < 0.050, groups were compared between each other, and the pair-wise P value reported; if the overall time effect was not significant, no further comparisons were done. All data in figures are presented as mean and standard deviation (SD) while data in the text are presented as slope, differences in slopes ± standard error (SE) or differences in least squares means (LSM) ± SE, as described.

## Results

### Propofol requirements

Throughout all experiments, the amount of propofol required to achieve loss of palpebral reflexes and orotracheal intubation was significantly higher following ACE (3.9±0.6 mg/kg IV) than following DEX (2.5±0.6 mg/kg) (P < 0.001).

### Body temperature

**Fig 2:**
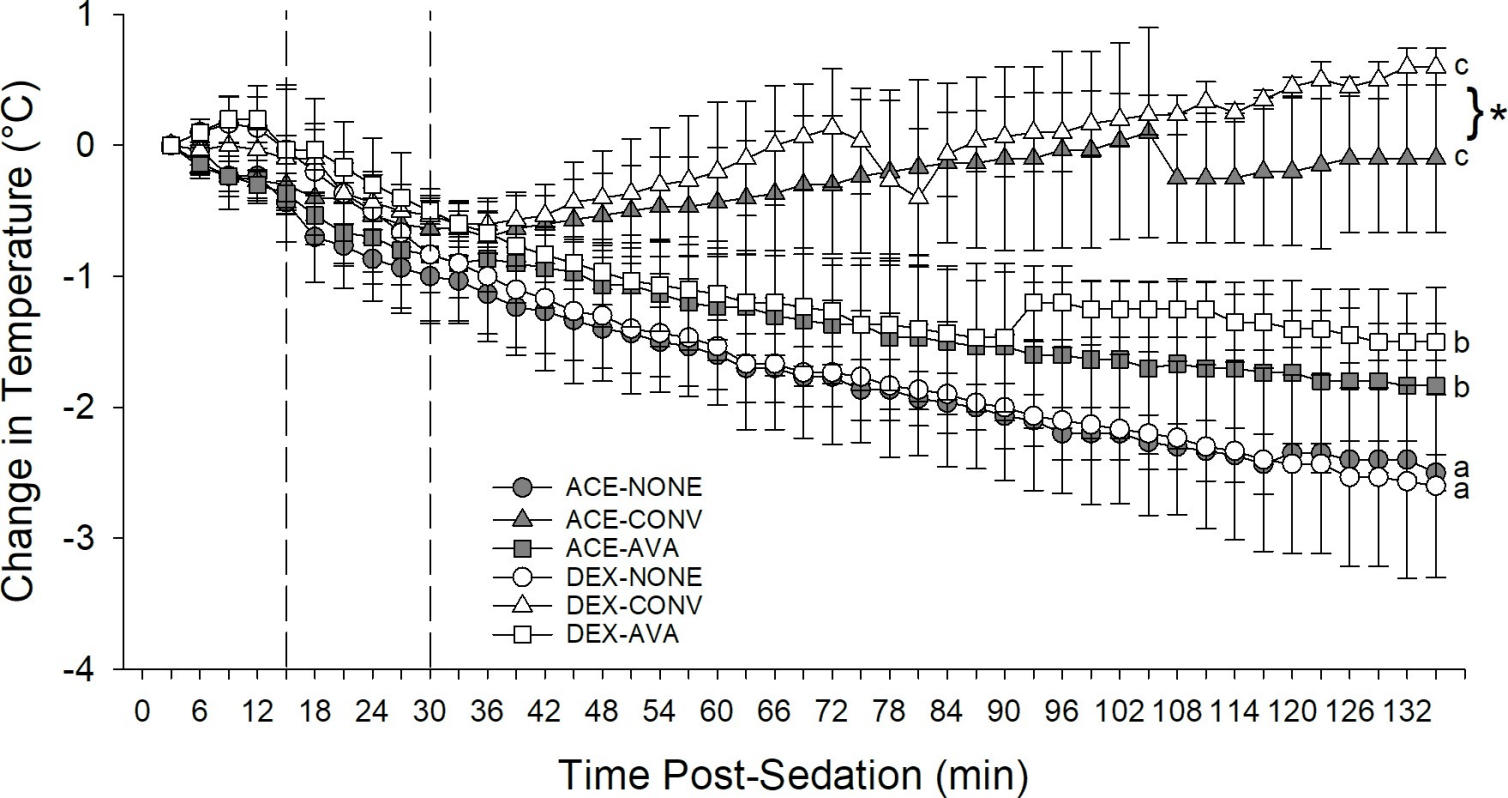
Change in rectal temperature following pre-medication, induction and inhalant anesthesia. Dogs were studied in six groups in a cross over design combining premedications and warming devices: acepromazine and no warming devices (ACE-NONE; gray circles), acepromazine and conventional warming devices (ACE-CONV; gray triangles), acepromazine and the AVAcore (ACE-AVA, gray squares), dexmedetomidine and no warming devices (DEX-NONE, white circles), dexmedetomidine and conventional warming devices (DEX-CONV, white triangles), and dexmedetomidine and the AVAcore (DEX-AVA, white squares). The absolute change in temperature from baseline showed three significantly different temporal patterns: T0-T15 minutes following premedication, T15-T30 minutes following propofol induction, and >T30 minutes following induction into anesthetic maintenance (all P < 0.05, differences indicated by dotted lines). Overall, when all time points and warming treatments were combined, ACE had significantly larger decreases in body temperature versus DEX, mainly attributable to significant differences at >T30 minutes (both P < 0.05). When ACE and DEX groups were considered separately, the slopes of the temperature changes differed between all warming treatments (NONE, CONV, AVA) despite premedication (significant differences between groups depicted by different lowercase letters; all P < 0.05). When warming treatment groups were analyzed separately, at >T30 minutes no significant differences in slopes were found between ACE and DEX within the NONE and AVA treatments but the DEX-CONV group had a more positive slope versus the ACE-CONV group (*P < 0.05). Data are presented as mean ± SD.

All dogs successfully underwent all treatments. However, in the DEX-CONV and ACE-CONV groups, one dog was removed at 72 minutes and 103 minutes, respectively, due to overheating. When all warming treatments and premedications were combined, the absolute change from baseline in rectal temperature increased significantly from T0-T15 (slope ± SE: 0.0275 ± 0.0114°C, P < 0.0138), the temperature change was not significant (slope ± SE: -0.006 ± 0.005°C, P = 0.1763) in the middle period (T15-T30 minutes) and the temperature decreased in the last period (>T30 minutes) (slope ± SE: -0.008 ± 0.001°C, P < 0.001); the three time periods were subsequently considered separately when applicable (Fig 2).

When all warming treatments and time points were combined, the decrease in rectal temperature from baseline was significantly greater in ACE than DEX groups (mean difference ± SE: -0.195±0.056°C, P = 0.001) (Fig 2). When the ACE and DEX groups were further considered separately, from T0-T15 minutes following premedication and between T15-T30 minutes, there were no differences in the slope of temperature changes between warming treatments in either the ACE or DEX groups (all P > 0.050) (Fig 2). However, at >T30 minutes, the slopes of the temperature change (± SE) of all three groups administered DEX (DEX-AVA versus DEX- CONV: -0.01683 ± 0.001175°C, DEX-AVA versus DEX-NONE: -0.007089 ± 0.001136°C, DEX-CONV versus DEX-NONE: 0.02392 ± 0.001114°C) differed significantly from each other (all P < 0.001) with the slope of the DEX-NONE group being most negative and DEX-CONV slightly positive (Fig 2). Similarly, at >T30 minutes, the slopes of the temperature change (± SE) of all three groups administered ACE (ACE-AVA versus ACE-CONV: -0.01354 ± 0.001026°C, ACE-AVA versus ACE-NONE: 0.004487 ± 0.001012°C, ACE-CONV versus ACE-NONE: 0.01803 ± 0.001053°C) differed significantly from each other (all P < 0.001) with the slope of the ACE-NONE group being most negative and ACE-CONV slightly positive (Fig 2).

At >T30 minutes, when warming device groups were analyzed separately, the slopes of the temperature change did not differ between ACE and DEX in the NONE and AVA groups (all P > 0.050) (Fig 2). However, in the CONV group, both ACE and DEX had slightly increased temperature changes, and DEX had a significantly faster increase in slope than did ACE (mean difference ± SE: -0.00249 ± 0.00866°C, P = 0.0045) (Fig 2).

### Anesthetic recovery times

There were no significant differences between premedications (ACE versus DEX; LSM ± SE) in time to tracheal extubation (10.75 ± 1.77 versus 11.25 ±1.77 min, P = 0.8121), sternal recumbency (15.59 ± 1.65 versus 13.30 ± 1.65 min, P = 0.4885) or standing (18.88 ± 1.91 versus 15.90 ±1.91 min, P = 0.4468) (Table 1). There were no significant differences between heating devices (NONE versus CONV versus AVA; LSM ± SE) in time to tracheal extubation (12.42 ± 3.78 versus 13.23 ± 5.02 versus 7.34 ± 2.47 min, P = 0.1014), sternal recumbency (14.87 ± 3.04 versus 17.71 ± 3.68 versus 10.76 ± 2.45 min, P = 0.3943) or standing (14.24 ± 2.85 versus 24.09 ± 3.01 versus 13.84 ± 2.72 min, P = 0.1255) and there was no relationship between the final rectal temperature prior to discontinuation of inhalant and tracheal extubation (P = 0.4937), sternal recumbency (P = 0.6283) or standing (P = 0.1570) (Table 1).

**Table 1:** Times to tracheal extubation, sternal recumbency and standing (minutes) following inhalant anesthesia and final rectal temperatures (°C) in each premedication-treatment group.

Group	Tracheal extubation	Sternal recumbency	Standing	Rectal temperature
ACE-NONE	15.0±3.0	17.7±2.9	19.3±2.3	35.7±0.6
ACE-CONV	9.3±6.7	18.0±11.0	23.3±11.7	38.2±1.0
ACE-AVA	8.3±3.8	11.3±4.7	15.7±5.9	36.1±0.3
DEX-NONE	14.3±3.1	14.7±3.5	16.7±2.5	35.4±0.4
DEX-CONV	10.7±6.7	13.7±5.9	15.0±6.2	38.7±0.5
DEX-AVA	8.3±5.1	11.3±7.4	15.3±10.0	36.3±0.6

### Arterial blood pressure and heart rate

**Fig 3:**
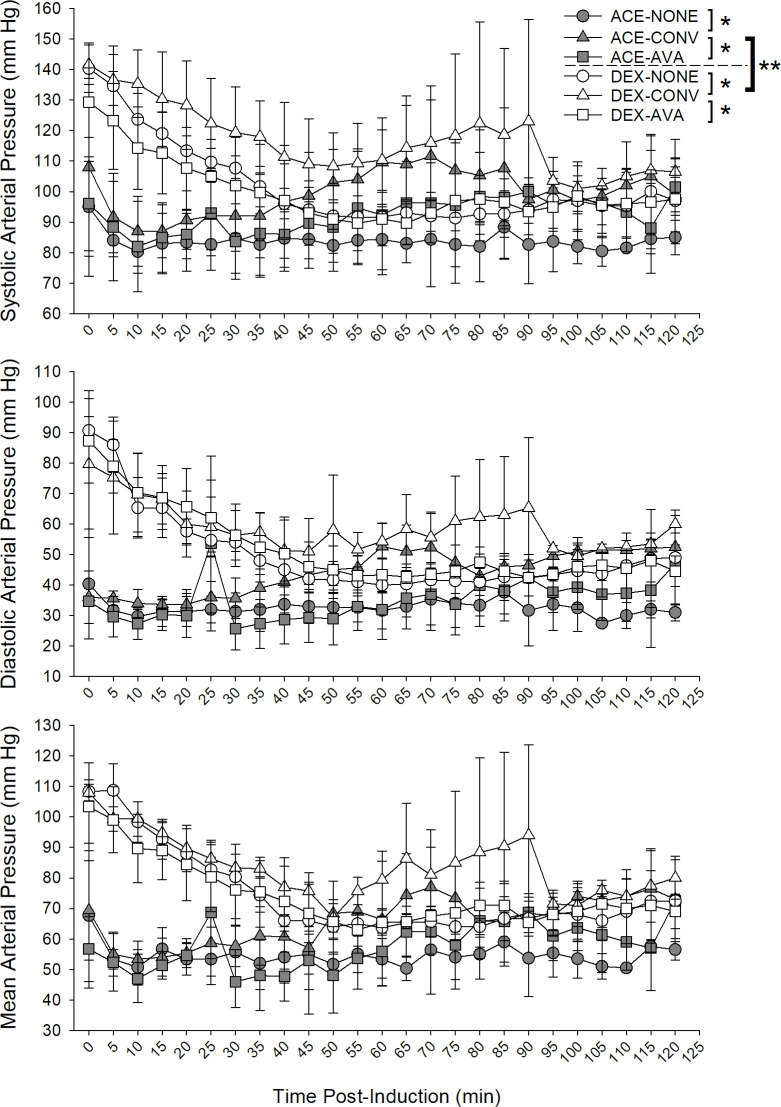
Effects of pre-medication and warming device on systolic (SAP), diastolic (DAP) and mean (MAP) arterial pressures in dogs following pre-medication, induction and inhalant anesthesia. Dogs were studied in six groups in a cross over design combining premedications and warming devices: acepromazine and no warming devices (ACE-NONE; gray circles), acepromazine and conventional warming devices (ACE-CONV; gray triangles), acepromazine and the AVAcore (ACE-AVA, gray squares), dexmedetomidine and no warming devices (DEX-NONE, white circles), dexmedetomidine and conventional warming devices (DEX-CONV, white triangles), and dexmedetomidine and the AVAcore (DEX-AVA, white squares). When all time points were considered, dogs administered ACE had significantly lower SAP, DAP, and MAP compared to DEX administration despite the warming treatment group (**P < 0.05). Overall, the ACE-CONV group had higher SAP, DAP and MAP compared with the ACE-NONE and ACE-AVA groups, and the DEX-CONV group had higher SAP, DAP, and MAP compared with the DEX-NONE and DEX-AVA groups, respectively (*P < 0.05).

**Fig 4:**
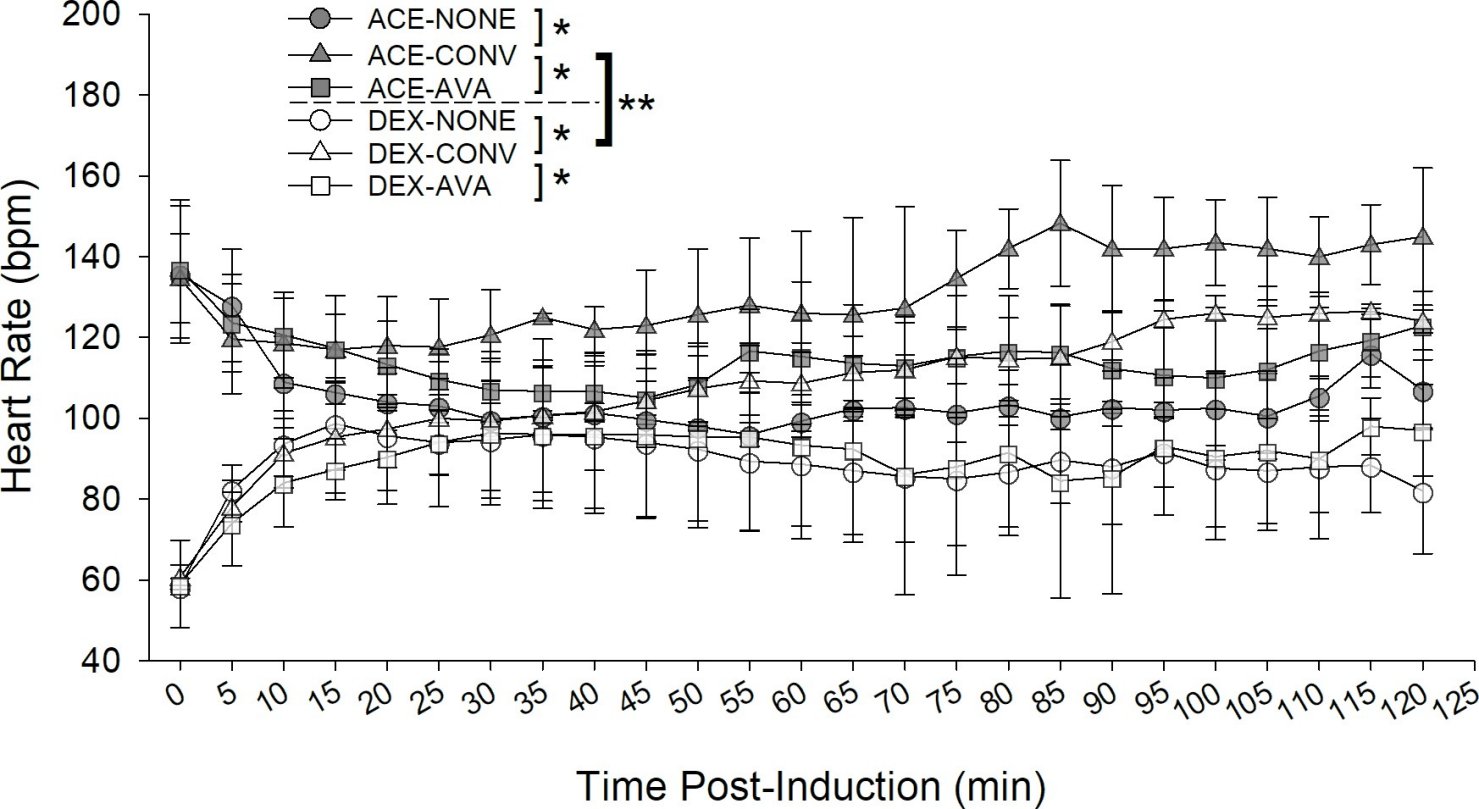
Effects of pre-medication and warming device on heart rate (HR) assessed by pulse oximetry in dogs following pre-medication, induction and inhalant anesthesia. Dogs were studied in six groups in a cross over design combining premedications and warming devices: acepromazine and no warming devices (ACE-NONE; gray circles), acepromazine and conventional warming devices (ACE-CONV; gray triangles), acepromazine and the AVAcore (ACE-AVA, gray squares), dexmedetomidine and no warming devices (DEX-NONE, white circles), dexmedetomidine and conventional warming devices (DEX-CONV, white triangles), and dexmedetomidine and the AVAcore (DEX-AVA, white squares). When all time points were considered, dogs administered ACE had significantly higher HR compared to DEX administration despite the warming treatment group (**P < 0.05). Overall, the ACE-CONV group had higher HR compared with the ACE-NONE and ACE-AVA groups, and the DEX-CONV group had higher HR compared with the DEX-NONE and DEX-AVA groups, respectively (*P < 0.05).

Arterial blood pressures (SAP, DAP, MAP; Fig 3) and heart rate (HR; Fig 4) were assessed to estimate degree of vasodilation or vasoconstriction associated with ACE or DEX. When all time points were considered, dogs administered ACE had significantly lower (reported in LSM ± SE) SAP, DAP, and MAP and higher HR compared to DEX administration despite the warming treatment group (SAP: 91.84 ± 2.47 versus 106.22 ± 2.47 mm Hg; DAP: 37.08 ± 1.18 versus 53.93 ± 1.18 mm Hg; MAP: 59.29 ± 0.68 versus 77.68 ± 0.70 mm Hg; mmHg; HR: 116.06 ± 6.23 mm Hg versus 95.19 ± 6.23 beats per min, all P< 0.001) (Figs 3 and 4). These effects were mainly attributable to differences immediately following induction up to minute 35 (SAP), 50 (DAP), 45 (MAP), and 25 (HR). Overall, the ACE-CONV groups had higher SAP, DAP and MAP and higher HR compared with the ACE-NONE (all P < 0.001) and ACE-AVA groups (P = 0.003, P < 0.001, P < 0.001, respectively; HR: P < 0.001) (Figs 3 and 4). Similarly, the DEX-CONV groups had higher SAP, DAP and MAP and higher HR compared with the DEX-NONE (all P < 0.001) and DEX-AVA groups (P < 0.001, P = 0.001, P < 0.001, respectively; HR: P < 0.0001) (Figs 3 and 4).

## Discussion

Consistent with our hypothesis, the AVAcore placed on both front paws at anesthetic induction reduced hypothermia associated with general anesthesia after 30 minutes compared to dogs with no warming devices placed, despite premedication with ACE or DEX. Although the AVAcore offers an easily applied technique to mitigate temperature changes in dogs, conventional warming methods (circulating water blanket and forced air warmer) were more effective in reducing the temperature decline and maintaining blood pressure. Overall, dogs sedated with ACE had more significant decreases in body temperature, SAP, DAP and MAP; decreases in temperature following DEX-CONV were less than for ACE-CONV. Although warming devices differed in the efficacy in maintaining body temperature during inhalant anesthesia, anesthetic recovery times did not differ between any premedication or treatment group.

In the present study, consistent with other reports in dogs [14], the slopes of the change in rectal temperatures were slightly positive immediately following premedication (T0-T15) and decreased significantly over time beginning 15 minutes following induction with propofol (>T30). However, due to large data variability in the present study, a statistically significant temperature decrease immediately following propofol induction (T15-T30) was not appreciated in our limited study; a larger sample size may have yielded other results. A higher propofol dose was required in the ACE group for endotracheal intubation, which may have attributed to greater degrees of vasodilation and temperature changes associated with ACE versus DEX groups. However, physiologic effects associated with propofol quickly diminish following induction [26]. Thus, prolonged effects of propofol on study outcomes, especially at later time points such as >T30, are likely minimal. Although warming technique had no effect during the first 15 minutes following anesthetic induction, the NONE, AVA and CONV groups significantly differed from each other afterwards, suggesting that the loss of thermoregulatory mechanisms and redistribution of blood flow immediately following anesthetic induction may be difficult to overcome, although efforts to maintain normothermia become more efficacious in later time periods.

Although the AVAcore did not maintain body temperature as well as the conventional devices, it did mitigate hypothermia to a degree and may offer unique advantages such as application to a nonintrusive, non-truncal site, particularly if an abdominal procedure is planned. Additionally, it requires only a commercial warm water pump; there are no forced air blankets which may propel particulates into the environment and can be frequently soiled with bodily fluids and scrub solutions if placed near the surgical site. In addition, significant side effects such as extreme body temperature swings due to overwarming or thermal burns are unlikely with the AVAcore since initial vasoconstriction regulates heat delivery to the body core but as the core warms, peripheral vasodilation occurs to dissipate heat, although these effects may be affected by general anesthesia. Although we did not observe any obvious epidermal lesions, we cannot confirm the complete absence of thermal burns and additional safety evaluation such as skin biopsies to detect subclinical changes should be performed.

We speculated that choice of premedication may affect warming technique efficacy based on their unique vasoactive properties. Although vascular tone was not directly measured in this study, blood pressure, and more specifically DAP [27–30], was used as an index of vasoactivity. Systemic blood pressure results from complex interactions between preload, afterload, myocardial dysfunction, heart rate, and vascular resistance, among other physiologic mechanisms. Although other factors apart from vascular resistance can affect DAP, diastolic values were used as a tool to indicate vasodilation, although the precise degree of vasodilation or vasoconstriction cannot be confirmed. ACE administration resulted in lower SAP, DAP and MAP blood pressures with likely compensatory increases in heart rates when compared with DEX in our dogs; effects mainly seen in the first 30–50 minutes following anesthetic induction. However, minimal differences in thermoregulation between ACE and DEX within a given warming device group were observed for the entirety of the study, suggesting that both AVA and CONV warming devices improved IPH despite vascular state, with CONV devices having more favorable outcomes.

Uncontrolled hypothermia may result in impaired hemostasis, reduced drug metabolism, prolonged anesthetic recovery, increased post-operative sympathetic responses and hyperglycemia, pronounced nausea and vomiting, greater wound infection rates, shivering, and increased oxygen consumption [3, 7, 8, 31–33]. Thus, avoiding IPH would be expected to improve patient care and concurrent morbidities. Although most of these physiologic measures were not studied here, no differences in recovery times were detected despite premedication agent or warming device. We speculate that substantial sedative or cardiovascular effects of ACE and DEX were largely diminished prior to recovery as blood pressure and heart rate changes associated with premedication were minimally detected by ~30–50 minutes following premedication. It is possible that recovery times at shorter or longer time points may differ as premedication effects may be more pronounced at early time points or body temperatures may have more profound effects as inhalants reach equilibrium.

Other limitations exist apart from those previously discussed. This pilot investigation consisted of a very small sample size which increases risk of both Type I and Type II errors. Although done in conjunction with a statistician in a cross over design to improve statistical power, larger investigations further defining the utility of the AVAcore to minimize IPH and potential usefulness during rewarming are required since individual variation may further affect the data. In addition, one dog in each CONV group was removed prior to study end as the upper limit of body temperature evaluation was reached, further reducing numbers. Evaluation using other warming device combinations such as placement of a warm water blanket underneath the dog with the AVAcore on the forelimbs or AVAcore placement on all four limbs would also give additional insights into its effectiveness. Furthermore, presence of no tissue damage must also be further confirmed in future studies.

## Conclusions

Although conventional warming techniques provided the most stable thermoregulation in normal anesthetized dogs, the AVAcore device applied to the forelimbs also moderated decreases in body temperature associated with general anesthesia despite premedication, providing an additional warming technique in dogs.

## Supporting information

S1 File (XLSX)
